# APOLO-Bari, an internet-based program for longitudinal support of bariatric surgery patients: study protocol for a randomized controlled trial

**DOI:** 10.1186/s13063-016-1246-z

**Published:** 2016-03-01

**Authors:** Eva M. Conceição, Paulo P. P. Machado, Ana Rita Vaz, Ana Pinto-Bastos, Sofia Ramalho, Cátia Silva, Filipa Arrojado

**Affiliations:** School of Psychology, University of Minho, Campus Gualtar, 4710-037, Braga, Portugal

**Keywords:** Bariatric surgery, Internet intervention, Eating behaviors, Post-surgery interventions, Weight regain

## Abstract

**Background:**

Despite evidence of successful weight loss for bariatric surgery patients, some patients experience considerable weight regain over the long term. Given the strong association between post-surgery health behaviors and outcomes, aftercare intervention to address key behaviors appears to be a reasonable relapse-prevention strategy. As the burden of obesity rates increases in healthcare centers, an internet-based program appears to be a reasonable strategy for supporting bariatric surgery patients in the long term. The primary purpose of the current project is to develop and test the efficacy and perceived utility of APOLO-Bari.

**Methods/design:**

This study is a randomized control trial, which will be conducted in two hospital centers in the North of Portugal; it includes a control group receiving treatment as usual and an intervention group receiving the APOLO-Bari program for one year in addition to treatment as usual. A total of 180 male and female participants who underwent bariatric surgery (gastric sleeve or gastric bypass surgery) for 12 to 20 months will be recruited. Both groups will complete a similar set of questionnaires at baseline, every 4 months until the end of the intervention, and at 6 and 12 months follow-up. Assessment includes anthropometric variables and psychological self-report measures. The primary outcome measure will be weight regain measured at the end of treatment, and at 6 and 12 months follow-up. The secondary aims are to test the cost-effectiveness of the intervention and to investigate psychological predictors and trajectories of weight regain. APOLO-Bari was developed to address the weight regain problem in the bariatric population by offering additional guidance to bariatric patients during the postoperative period. The program includes: (a) a psychoeducational cognitive-behavioral-based self-help manual, (b) a weekly feedback messaging system that sends a feedback statement related to information reported by the participant, and (c) interactive chat sessions scheduled with a trained psychologist in the field.

**Discussion:**

APOLO-Bari may play an important role in broadening therapeutic reach to bariatric patients who would not otherwise have continuous support, with important implications for public health treatment.

**Trial registration:**

Current Controlled Trials: ISRCTN37668662.

## Background

### Bariatric surgery and its effect on weight loss

Long-term follow-up studies of bariatric surgery patients under treatment for severe obesity suggest that although most patients will achieve successful weight loss and weight maintenance [1], a significant subgroup of patients regain weight in the long term [2]. Despite different studies reporting significantly different values, researchers estimate that approximately 30 % of patients experience long-term weight regain, although this value may be as high as 97 % [2]. Recent studies report that excessive weight regain (i.e., more than 25 % of the weight lost after surgery) is experienced by approximately 37 % of patients [3]. Weight regain appears to be a particular concern between 12 and 24 months after surgery [1, 4] because this subgroup of patients presenting weight recidivism may find that the initial improvement of obesity-related comorbidities is mitigated [2, 5].

Although weight regain could result from physiological or anatomical factors, it is highly likely to result from the postoperative reemergence of maladaptive eating and lifestyle behaviors [6]. The greatest challenge for these patients may stem from a failure to embrace required lifestyle changes [7]. Other factors associated with poor outcomes include a lack of physical activity, low self-esteem, depression, or a low number of follow-up medical visits and postoperative self-monitoring behaviors [8]. Furthermore, a growing body of literature suggests that poor outcomes can largely be predicted by the post-surgery reemergence or development of dysfunctional eating patterns [9], such as patterns of uncontrolled or disordered eating [10], binge eating [11], grazing [12], or unhealthy food choices [13]. Whereas anatomical factors may necessitate additional surgical intervention, the management of psychological or behavioral factors is primarily non-surgical, and the continuous monitoring of key factors is crucial for early detection of behaviors that place patients at risk of weight regain. In this context, aftercare interventions that aim to monitor risk behaviors systematically and improve adjustments to healthy lifestyles appear to be a reasonable relapse-prevention strategy [9].

### Strategies to optimize weight outcomes

Psychological or support group interventions have been suggested to promote weight loss and weight control after surgery, physical activity, and adherence to recommendations, resulting in less maladaptive eating behaviors and better psychosocial functioning [6].

Pre-operative interventions appear to have beneficial effects, including a reduction in body weight and anxiety or depressive symptoms [14], binge eating, and maladaptive eating behavior [15] before surgery. However, their impact on postoperative weight loss is questionable, as increased weight loss has not been associated with these interventions one year after surgery [14, 16].

Other studies have investigated the role of postoperative psychological interventions and have shown promising results in reducing binge eating and eating behaviors [17, 18], supporting weight loss or reversing the tendency to regain weight [17, 19]. Moreover, a review and meta-analysis of postoperative behavioral management suggested that such interventions might optimize weight loss following surgery, although the results of some studies were not statistically significant. The conclusions of such studies should be interpreted with caution because they included small and heterogeneous samples [6].

The importance of post-surgery interventions has also been related to increased compliance with treatment requirements [20], enhanced motivation and confidence [21], and increased awareness of problematic eating patterns [22]. Moreover, greater weight loss was further associated with attending post-surgery meetings [21, 23–25].

Behavioral interventions appear to hold promise in inducing weight loss, but there is a lack of well-controlled studies [26, 27], and most interventions require face-to-face sessions. Furthermore, given the genuine effect of bariatric surgery on weight loss that most patients experience immediately after surgery [14, 28], research is needed to investigate the role of psychobehavioral interventions as an early prevention strategy for weight regain in the long term.

### Importance of using internet-based tools

With the demand of bariatric surgeries likely to continue its upward trend in the coming decades, the question remains as to how to continuously monitor and support these patients, particularly in the long term, in an environment with limited resources and rising rates of bariatric surgeries [29]. Another major difficulty is the accessibility of specialized treatment centers, especially in areas with poor medical infrastructure. As obesity rates place an increasing burden on healthcare centers, the development of alternative healthcare delivery strategies is gaining attention [30].

Internet-based programs offer several advantages, including strong accessibility, portability [31], easier dissemination and a reduced burden associated with clinical visits [32]. Furthermore, such programs overcome the problem of isolation of users and allow access to relevant information when needed, which results in reduced costs for healthcare services and for users [30, 33]. New technologies have been used to deliver psychological interventions with promising results for the treatment of binge eating disorder among obese and overweight patients [34] and overweight children and adolescents, and to help bulimia nervosa patients control their eating [35]. Despite the potential of new technologies for behavioral assessment and interventions in bariatric surgery [32], to date, no study has tested the effectiveness and feasibility of new technologies to prevent weight regain and optimize weight loss in bariatric patients. Nonetheless, much of what has been learned about effective strategies to support weight loss and weight maintenance can be incorporated into such interventions. Important strategies to be incorporated into internet-based interventions include self-monitoring to track a patient’s key behaviors and personalized feedback on their progress. These interventions also represent a valuable communication channel between patients and health professionals [32].

APOLO-Bari was developed to address the weight regain problem in the bariatric population by offering additional guidance, a monitoring system, clinical support, and a psychoeducational program to bariatric patients during the postoperative period. Such a program would also bridge the gap between the patient and clinical settings in a cost-effective format. APOLO-Bari is intended to work as an adjunct intervention to treatment as usual while investigating prospective predictors of weight regain. Although some commercial programs have been offered by manufacturers of bariatric surgery devices [32], to the best of our knowledge, this project is the first that aims to develop a specific, long-term internet-based program for bariatric surgery patients and to test its short- and long-term efficacy, utility and feasibility, and effectiveness as an aftercare strategy.

### Intervention strategy of APOLO-Bari – the rationale

After initial and dramatic weight change, patients can consolidate and build on their new and healthy lifestyles during the postoperative period. Handling post-surgery demands implies the need for behavioral modifications to engage in a healthy lifestyle and to comply with prescribed nutrition guidelines, especially at later follow-up times. However, as the follow-up time increases, the number of consultations with the medical team invariably decreases, resulting in a gap between health services and patients, particularly patients who might not otherwise have access to specialized treatment because of a lack of available resources outside of urban centers. With less frequent medical appointments over time, problematic behaviors might be overlooked and might not be clinically addressed, resulting in eventual weight regain.

The novelty and strength of the program APOLO-Bari are based on the ability to monitor key patient features related to outcomes continuously, to support and guide patients continuously, and to provide cognitive-behavioral-based support during the follow-up period, either to improve results or prevent poor outcomes. This approach attempts to narrow the gap between clinical services and patients by providing long-distance support to a large number of patients simultaneously, without the need for face-to-face sessions.

However, the disadvantages of internet-based programs have been noted; hence, the feasibility of the widespread utilization of such programs is called into question: weak adherence through the minimal use of internet resources, high rates of attrition, less education and less initial weight loss have been associated with dropout rates. In contrast, various studies have shown a positive association between log-in frequency and weight loss [30]. To overcome some of these difficulties, APOLO-Bari includes interactive tools that are designed to enhance adherence to the program, such as a feedback messaging system that is based on the patient’s input and a privileged channel (chat sessions) to contact a professional directly with specific concerns.

The APOLO-Bari components described here are not intended to replace necessary treatment or individual care targeting more complex problems when necessary (e.g., binge eating, bulimia nervosa, depression). Rather, the system enables the systematic monitoring of risk behaviors and refers patients to more intensive care when needed. APOLO-Bari is intended to be an adjunctive intervention to treatment as usual to improve outcomes by preventing weight regain and alerting the research and clinical team of patients who are at increased risk for weight regain. The major program objective is to prevent long-term weight regain through:

1. Providing self-help for the education and training of relevant skills for the postoperative period (self-help manual);
2. Promoting healthy and adaptive eating patterns (feedback messaging system);
3. Ensuring the early detection of risk behaviors (feedback messaging system);
4. Bridging the gap between patients and clinical settings while allowing continuous and direct contact with bariatric surgery specialists (chat sessions);
5. Improving compliance with daily behavioral adjustments (all components involved).

### The APOLO-Bari program and its three components

The program includes three components: (i) a self-help manual, which is psychoeducational cognitive-behavioral based and includes information on different topics relevant to weight regain prevention, as well as tasks that are related to different topics; (ii) a weekly feedback monitoring system with immediate feedback responses to assess risk behaviors, send a feedback statement related to the information reported by the participant and his or her historical reports, and provide reinforcement and guidance when a problem is detected; and (iii) direct contact with a trained psychologist in the field through scheduled interactive chat sessions in which participants can ask questions.

#### The self-help manual

The self-help manual includes an initial chapter that provides general information on obesity and bariatric surgery followed by 12 different psychoeducational and CBT-based chapters. A new chapter will be available each month. Participants can choose the chapter that best fits their needs each month. There is no pre-established sequence of chapters, as each chapter addresses different skills that do not rely on previous knowledge. One exception is the first chapter, which is the same for every participant, given its motivational nature. To complete the chapter successfully, participants must submit information on the respective tasks. Completed chapters remain open for future consultation. Tangible lifestyle changes that will be practiced during intervention will be:

1. Having regular and planned eating patterns;
2. Have a daily protein intake between 60 g and 80 g.
3. Achieve at least 150 min/week of structured physical activity plus 5000–7499 steps per day; or take at least between 7500–9999 steps per day.
4. Having systematic self-monitoring strategies.

Table 1 presents the title and objectives of each chapter of the self-help manual. Further information regarding the contents of this self-help manual can be provided by the authors on request, for research and replication purposes.

**Table 1:** Chapter from the self-help manual component of APOLO-Bari

Chapter	Objectives	Description and lifestyle changes
General information: *obesity* and *bariatric surgery* Note: Provided in the open area of the homepage. All study participants will have access to this chapter after registering.	To educate about obesity and bariatric surgery as a long-term intervention treatment.	Different procedures and their post-surgery implications; Obesity, psychosocial impairments and maintenance mechanisms; The role of bariatric surgery as a weight loss treatment for obesity.
*Motivation for intervention and importance of monitoring weight*	To highlight individual responsibility and the need for long-term lifestyle modifications; To educate about self-monitoring and regular self-weighing.	How to use the program; The top ten recommendations of your bariatric surgeon; How weight varies after surgery and in the long term; Why and how to control your weight.
*Healthy eating behavior*	To promote adequate eating behaviors; To educate about strategies to maintain a regular eating plan; To educate about the importance of the different nutrients and how to include them in a daily eating plan.	Nutritional changes and deficits after bariatric surgery; Rules for a healthy eating patter after bariatric surgery; Nutritional pyramid for post-bariatric patients; Signs of problems with your eating behaviors; Getting to know the different nutrients; How to read food labels;
*Stress management and problem solving*	To learn problem-solving strategies; To identify problematic situations; To anticipate and provide training on adequate coping strategies.	Different types of stress; Symptoms of stress after bariatric surgery; Steps in problem solving; Identify and deal with problematic situations; Stress management techniques;
*Physical activity and physical exercise*	To promote an active lifestyle; To educate about the importance of physical activity after surgery and to identify strategies to increase physical activity.	Benefits and myths of an active lifestyle after bariatric surgery; How to begin and increase physical activity; Use a pedometer and enhance your motivation; Sedentary versus active attitudes; Structured physical exercise: what is it and what is it not? Identifying and managing barriers for regular physical activity.
*Goals and expectations*	To adapt outcome expectations in different areas of life and throughout the weight loss process; To identify the individual meaning of success and failure.	Unrealistic expectation regarding bariatric surgery; Facts about weight loss after bariatric surgery; Expected weight loss after surgery; Identify non-weight related success outcomes after surgery; Establish ‘SMART’ goals and rewards.
*Emotions, thoughts and eating*	To understand the relationship between emotions and eating; To cope with anxiety and mood changes after surgery.	Change your thoughts to change your behavior; Think about your thinking – cognitive bias; Consequences and characteristics of emotional eating; Guidelines to deal with emotional situations; Physical hunger vs emotional hunger; Guidelines for challenging situations.
*Self-concept and self-esteem*	To understand the main aspects of incorporating a developing self-worth system; To identify low self-esteem negative thoughts.	Identify self-strengths, weaknesses, achievements and talents; Challenging low self-esteem negative thoughts.
*Body image*	To address common body image problems related to weight changes or extra hanging skin; To inform about aesthetic surgery; To educate about body checking and ‘feeling fat’.	Body image and (un)realistic expectations after bariatric surgery; Excessive skin and plastic surgery; Work on non-weight related positive qualities; Accepting less positive body aspects; Biased assumptions about the importance of body image; Body checking and ‘feeling fat’.
*Eating behavior problems*	To identifying persistent eating problems; To identify and cope with triggers for problematic eating behaviors (e.g., emotional eating, binge eating, loss of control, grazing, dumping or plugging).	Persistent eating problems: binge eating and loss of control; excessive eating; grazing; night eating; restriction; sneak eating; chewing and spitting; food choices and restaurant choices; Gastrointestinal problems: vomiting; dumping syndrome and plugging; Reducing calories changing the way you eat; Monitoring eating behaviors and identifying trigger for problematic eating.
*Relationships and interpersonal difficulties*	To cope with new emerging problems; To provide training on assertiveness and interpersonal skills.	Challenges experienced with social interactions; Make social cues work for you; Techniques to express yourself assertively.
*Social support and significant others*	To promote an adequate social support system; To identify helpers or those who might create challenging situations.	Communicating with significant others; Pregnancy and contraception; Changes experienced by your significant other; Assessing your current social support: Defining how others can help you.
*Relapse prevention*	To explain the difference between a misstep and a relapse; To alert for common problems associated with weight regain in the long term; To identify risk behaviors and coping strategies.	How to keep motivated; Anticipate problematic situations; Relapse versus lapse; Create a relapse-prevention plan.

SMART = Specific, Measurable, Attainable/Achievable, Relevant, Timely

#### The weekly feedback monitoring system

This system consists of a self-monitoring internet-based tool that allows patients to regularly input monitoring information in response to pre-programmed questions. Patients respond weekly to a brief standardized questionnaire that addresses the status of various key features described in the measures section, maintaining a log of the individual process. Based on the response to items that assess grazing, binge eating, and overeating in the feedback messaging system, the computer program immediately evaluates the input information and compares the current status with the preceding week in order to send a tailored feedback message. Based on a pre-programmed algorithm, the server automatically selects a message from a feedback message pool to be sent immediately after the participant submits the feedback messaging system information. Each of the three risk behaviors is divided into a symptomatic and non-symptomatic range and, each week, the system determines the pattern of change since the previous week: whether the participant improved (from symptomatic to non-symptomatic), remained unchanged and non-symptomatic (positive), remained unchanged and symptomatic (negative), or deteriorated (from non-symptomatic to symptomatic). A symptomatic state is indicated when a patient reports at least one of the key behaviors one or more times in the previous week. For this purpose, a comprehensive pool of feedback messages was formulated and integrated into the program. A total of 977 messages were pre-prepared, corresponding to the possible combinations of eating behaviors (3) and the possibilities of change (4) (4 × 4 × 4). For each combination, 10 to 15 messages were formulated, to avoid the repetition of information. Furthermore, 112 messages were formulated for assessments in which no information was provided for the previous week (missing data). For each possible combination of symptomatic and non-symptomatic risk behavior, 14 different messages were formulated (2 × 2 × 2 × 14). One additional reminder message was included to motivate participants who missed a week’s assessment. Each message is different and includes (a) praise for their progress, (b) a reinforcement statement, and (c) alerts or advice for alternative behavior in the case of deterioration. Table 2 presents examples of feedback messages for different patterns of change.

**Table 2:** Examples of feedback messages for different patterns of change used in the algorithm for the feedback message system

Grazing	Binge eating	Overeating	Feedback message
n	n	d	Be aware! Your eating is becoming disorganized. Try to understand what is happening – that is the only way you can get back on track. Do not give up on yourself!
n	p	p	To reduce grazing, do not skip meals, and take time to sit down while you are eating. You’ll see the benefits soon!
i	i	n	We have noticed that you have reduced the frequency of some maladaptive eating behaviors. Try to focus your attention on overeating episodes. Do not keep leftovers, and limit the amount of food you have at home, especially those foods that make you feel out of control. You have every reason to keep investing in yourself.
d	d	p	People often eat in a compulsive way to cope with strong emotions, particularly when they are sad, hungry, or anxious. It is important to anticipate a list of alternative activities to distract yourself from eating in those situations. You may want to avoid going to the grocery store to limit the amount of food available.
p	n	p	Be aware of when binge eating episodes occur. Awareness allows the anticipation of difficult situations and the identification of triggers for these episodes. It also helps to understand the role of these episodes in your life (e.g., eating compulsively to cope with certain feelings or emotions, eating at the end of the day when feeling lonely or when you have a day off work). You can then find alternative strategies to anticipate such difficult situations. Go for it!
p	p	p	We are happy that you have been able to keep a regular and adjusted eating pattern. Enjoy the possibility of having a healthy life, and you will have no problem maintaining a healthy weight.

*n* negative (symptomatic), *p* positive (non-symptomatic), *i* improved (from symptomatic to non-symptomatic), *d* deteriorated (from non-symptomatic to symptomatic)

The feedback messaging system will be open once a week. On a pre-determined day, the system will send an email to remind or invite the participant to respond to the feedback messaging system.

Furthermore, the server will send a motivational message directly when a participant has missed a weekly assessment or has not logged in for more than a month. The system will automatically send an email (‘case alarm’) to the research team when a participant reports symptoms that require additional clinical attention: (i) weight regain of >5 kg over the previous 3 months and (ii) the presence of any risk behaviors every week for at least 3 months. In such a case, the research team will contact the participant via email.

#### The chat sessions

Several chat sessions will be available every week based on the previous schedule. A participant who wants to ask specific personal questions can register for a pre-scheduled session and log in on the day and time determined. The specialist conducting the chat session is a master’s or PhD level psychologist with more than 3 years of clinical experience with pre- and post-bariatric patients. The psychologist assigned to the session will be online for 30 minutes to chat with registered participants. Patients are allowed to schedule a session every other week. All of the psychologists who are involved in the chat sessions are under frequent supervision at the University of Minho.

## Objectives of the study and expected outcomes

The specific objectives of this project are the following:

1. To test the short- and long-term efficacy of the program in preventing weight regain and promoting weight loss maintenance, promoting new eating behaviors, decreasing psychological distress, enhancing individual self-concept, and addressing other psychological outcomes.
2. To test the utility, feasibility, and perceived satisfaction with APOLO-Bari in terms of compliance with proposed tasks, log-in frequency, and direct evaluation of the satisfaction of participants. Participants with higher log-in frequency and compliance with monthly assessments are expected to report better weight outcomes, higher levels of satisfaction with weight loss and surgical treatment, and lower scores on the self-reported measures, thus reflecting better psychosocial functioning.
3. To study weight regain by exploring the temporal courses of eating-related features and predictors of weight regain. We expect to identify fluctuations in key behaviors that precede weight regain.

## Methods/design

### Design and procedures

This a randomized controlled trial (Current Controlled Trials: ISRCTN37668662), with a superiority design that includes two parallel groups: a treatment-as-usual control group and an intervention group, who receive both treatment as usual and the internet-based program. Consecutive participants will be recruited from two hospital centers in the North of Portugal. Information about the hospital centers involved can be found on the current controlled trials website for our trial registration number.

All participants will be contacted by phone or during their medical appointment and screened for the inclusion and exclusion criteria. Patients meeting these criteria will be invited for an orientation group session to inform them of the program and study procedures. The study will be introduced as a program to support bariatric patients remotely in the long term, which aims to monitor relevant variables and investigate patients’ perception of the utility of these interventions.

### Retention and incentives for the participants

The following strategies will be used to minimize attrition. First, there will be a financial incentive: the participants will receive a €20 voucher to use in a main retail chain store after completing the program. To be eligible for this reward, the participants must complete at least 70 % of their required assessments. Second, the participants will be asked for two contact phone numbers or email addresses at registration. Third, reminder emails will be sent every time a questionnaire or activity is available. Finally, an orientation session will be held before the beginning of the intervention.

### Informed consent and randomization

Those who agree to participate in the study will have a computer connected to the internet, and each participant will complete the informed consent form online and register in the program by creating a personal username and password. All participants will complete an initial short sociodemographic and clinical questionnaire (see details in the measures section). To ensure concealment of allocation, the APOLO-Bari system will then automatically randomize (computer-generating random numbers) the participants on a 1:1 basis, matching for age, sex, and surgery type.

After randomization, intervention group members will receive full access to the three components of the APOLO-Bari program in their personal accounts. The intervention will last for 12 months, and a 6-month period will follow, with no program access possible. During the intervention period, the system will record data on log-in frequency, task submission, and weekly monitoring from the intervention group.

The control group will only have access to general information on obesity and bariatric surgery and to the set of self-report measures at the different assessment times. The control group participants will be informed of the assessment times and the possibility of accessing the full program after the study is complete. Both groups will receive reminder emails every time a questionnaire or activity is available.

### Subjects

Participants (male and female) between 18 and 65 years of age who underwent bariatric surgery (sleeve gastrectomy and gastric bypass) at least 12 months and not more than 20 months previously will be recruited. This timeframe is used to anticipate the period when patients appear to be at risk of weight regain (12–24 months postoperatively) [2] and when they need help coping with lifestyle adjustments.

For the purpose of this study, patients who initially lost weight successfully after surgery will be included. We seek to exclude potential medical or surgical problems that are not related to lifestyle and psychological adjustment and that could be implicated in and limiting to the initial weight loss. Furthermore, given that our ultimate objective is to prevent weight regain, significant initial weight loss is required. Significant weight loss will be considered when the percentage of excessive weight loss is higher than 30. Furthermore, participants who present with significant weight regain (more than 15 % of the total weight loss) since the nadir weight at the time of registration will be excluded. Other specific exclusion criteria include the following:

1. No regular internet access;
2. Inability to read and understand Portuguese instructions;
3. Lack of willingness to have or create an email account;
4. Presence of an active psychiatric disorder (e.g., bipolar disorder, psychotic disorder, suicidal ideation, eating disorders);
5. Intake of weight-affecting drugs;
6. Concomitant weight loss treatment besides treatment as usual;
7. Pregnancy or lactation.

### Proposed sample size and power calculations

The sample size was computed by considering a 0.2 effect size, an alpha level of 0.05, a desired statistical power of 0.8, four predictors or measures and a 20 % dropout rate; this calculation suggested that a total of 180 participants should be recruited (90 in each group).

### Assessment

**Fig. 1:**
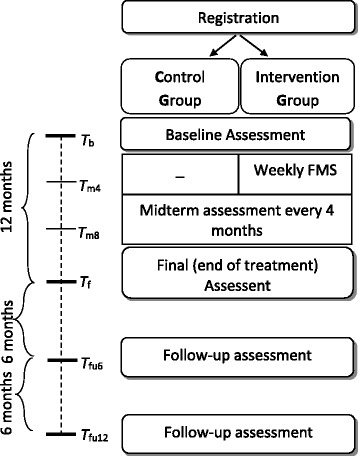
Flow chart of the assessment times of the study protocol. FMS, feedback messaging system; *T*_b_, baseline assessment_;_ *T*_m4_, 4-month midterm assessment; *T*_m8_, 8-month midterm assessment; *T*_f_, final (end of treatment) assessment; *T*_fu6_, 6-month follow-up assessment; *T*_fu12_, 12-month follow-up assessment

Figure 1 depicts the time flow and assessment times for this project.

#### Anthropometric measures

Participants will be instructed to report their weight based on a standardized assessment self-reported using the same scale on the same day of the week under the same conditions (the time of day and without clothes). The program will ask participants to report their weight once a month in one of their weekly assessments as part of their self-monitoring tasks. Both the intervention group and the control group will have their height and weight measured by the research team at baseline (*T*_b_), at 4- and 8-months midterm assessment (*T*_m4_, *T*_m8_), at the end of the intervention (final; T_*f*_), and at 6- and 12-months follow-up (*T*_fu6_, *T*_fu12_). The weight history of participants (e.g., highest and lowest weight since adulthood, pre-surgery weight, and lowest weight since surgery) will be self-reported.

#### Self-report questionnaires

##### Sociodemographic and weight history

This history assesses data on the demographic information (sex, age, and marital status) and relevant clinical variables (type of surgery, pre-surgery weight, lowest and highest weight before surgery, lowest weight achieved after surgery).

##### Clinical inventory

This inventory assesses the history of psychological/psychiatric disorders, current medications and other current psychiatric or psychological disorders, as well as alcohol use.

### Psychological self-report measures

Repetitive Eating Questionnaire [12]: This self-report questionnaire assesses a grazing-type eating pattern through 15 items, resulting in two subscales that reflect compulsive grazing and non-compulsive grazing subtypes.

Depression Anxiety Stress Scales [36]: This 21-item questionnaire generates subscales on depression, anxiety, and stress.

Eating Disorder – 15 [37]: This 15-item questionnaire assesses eating disorder cognition and behaviors, with subscales for weight and shape concerns and for eating concerns.

Negative Urgency Subscale [38]: This 12-item scale assesses an individual’s tendency to surrender to strong impulses, particularly when accompanied by negative emotions, such as depression, anxiety, or anger.

Influence of weight: Developed by the research team, this 12-item measure inquires about the influence of weight in different areas of life (e.g., intimate relationships, professional roles, self-esteem).

Satisfaction questionnaire: Developed by the research team, this 19-item questionnaire addresses the utility and feasibility of the program from the patient’s perspective.

All questionnaires have been used with this sample by the research team in previous studies, and show good internal consistency for use with bariatric surgery patients.

Table 3 presents a summary of the self-report measures used for each group at each assessment time.

**Table 3:** Timeline for the self-report measures

	Registration	Baseline (*T*_b_)	4-month midterm assessment (*T*_m4_)	8-month midterm assessment (*T*_m8_)	Final (end of treatment) assessment (*T*_f_)	6-month follow-up assessment (*T*_fu6_)	12-month follow-up assessment (*T*_fu12_)	Weekly
Sociodemographic weight history	X	–	–	–	–	–	–	–
Clinical inventory	–	X	X	X	X	X	X	–
Repetitive Eating Questionnaire [12]	–	X	X	X	X	X	X	–
Depression Anxiety Stress Scales [36]	–	X	X	X	X	X	X	–
Eating Disorder – 15 [37]	–	X	X	X	X	X	X	–
Negative Urgency Subscale [38]	–	X	X	X	X	X	X	–
Influence of weight	–	X	X	X	X	X	X	–
Satisfaction questionnaire	–	–	–	–	Intervention group	–	–	–
Eating and exercise behaviors (Table 4)	–	Control group	Control group	Control group	Control group	X	X	Intervention group

*X* available for both intervention and control group

### Weekly assessment

The weekly assessment includes three questions that will provide information for the feedback message and six other questions designed to monitor the relevant behaviors for this population. For the feedback message, the participants respond to the number of days in the previous week that they engaged in grazing, binge eating, or overeating. Definitions are provided based on the definitions proposed by Conceição et al. [12] for grazing, and by Fairburn et al. [39] for overeating eating and binge eating episodes. Other questions involve additional eating behaviors (e.g., skipping meals, the level of control over eating) and active or sedentary behaviors. Table 4 presents a list of the items used in the weekly assessment.

**Table 4:** Weekly assessment questionnaire for the feedback messaging system

Please respond to the following questions by referring to the previous week (7 days). During the past week…
1. How many days did you skip meals (for example, breakfast, morning snack, lunch)?
2. How many days did you graze or nibble on small or modest amounts of food throughout the day in a repetitive and unplanned manner?
3. How many days did you feel that you overate (for example, had a second helping, continued eating after satiation, overate without feeling hungry)?
4. How many days did you feel that you overate or could not resist eating because you were feeling anxious, nervous, or sad, or other emotions?
5. How many days did you feel that you lost control over what you were eating (for example, being unable to resist eating or to stop eating after you started)
a. On a scale from 1 to 5 (with 1 indicating very low and 5 indicating an extreme loss of control), to what extent did you feel a sense of loss of control during those situations?
6. How many days did you eat compulsively, losing control while eating an amount of food that was extremely large given the situation (for example, twice or more than what others would eat under the same situation)?
a. On a scale from 1 to 5 (with 1 representing very low and 5 representing an extreme loss of control), to what extent did you feel a sense of loss of control during those situations?
7. How many days did you walk for more than 15 minutes?
8. How many days were you involved in structured physical activity for more than 30 minutes, such as going to the gym, playing soccer, or swimming?
9. On average, how many hours per day did you spend sitting for any reason (for example, working, browsing the internet, driving, watching TV on the sofa)?
10. Did you have surgery to remove the extra hanging skin this week? Yes, Date__; No.

### Primary and secondary outcome measures

The primary outcome measure will be weight regain assessed at baseline (*T*_b_), end of treatment (*T*_f_), and follow-up (*T*_fu6_ and *T*_fu12_). Other weight outcome measures, such as variance in weight loss estimations, will be tested. The following formulas will be used to compute these outcomes [40]:

$$ \mathrm{Percentage}\ \mathrm{of}\ \mathrm{total}\ \mathrm{weight}\ \mathrm{regain} = \left(\frac{\mathrm{current}\ \mathrm{weight}-\mathrm{nadir}\ \mathrm{weight}}{\mathrm{weight}\ \mathrm{presurgery} - \mathrm{nadir}\ \mathrm{weight}}\right)\times 100; $$

$$ \mathrm{Percentage}\ \mathrm{of}\ \mathrm{excessive}\ \mathrm{weight}\ \mathrm{regain}=\left(\frac{\mathrm{current}\ \mathrm{weight} - \mathrm{nadir}\ \mathrm{weight}}{\mathrm{excess}\ \mathrm{weight}}\right)\times 100; $$

$$ \mathrm{Percentage}\ \mathrm{of}\ \mathrm{total}\ \mathrm{weight}\ \mathrm{loss}=\left(\frac{\mathrm{weight}\ \mathrm{presurgery} - \mathrm{current}\ \mathrm{weight}}{\mathrm{weight}\ \mathrm{presurgery}}\right)\times 100; $$

$$ \mathrm{Percentage}\ \mathrm{of}\ \mathrm{excessive}\ \mathrm{weight}\ \mathrm{loss}=\left(\frac{\mathrm{weight}\ \mathrm{presurgery} - \mathrm{current}\ \mathrm{weight}}{\mathrm{excess}\ \mathrm{weight}}\right)\times 100; $$

$$ \mathrm{Percentage}\ \mathrm{of}\ \mathrm{alterable}\ \mathrm{weight}\ \mathrm{loss}=\left(\frac{\mathrm{body}\ \mathrm{mass}\ \mathrm{index}\ \mathrm{presurgery} - \mathrm{current}\ \mathrm{body}\ \mathrm{mass}\ \mathrm{index}}{\mathrm{body}\ \mathrm{mass}\ \mathrm{index}\ \mathrm{presurgery} - 13}\right)\times 100\ . $$

Excess weight will be calculated based on metropolitan guidelines [41].

Secondary outcome measures will include behavioral and psychological outcome measures: the number of days engaging in maladaptive eating behaviors (such as grazing, binge eating, or overeating episodes) as well as the number of hours of sedentary and physical activities in the previous month. Psychological symptoms assessed with self-report measures will also be tested. Differences between the intervention group and the control group will be tested at all assessment times. Additionally, midterm secondary outcomes will be tested as predictors of weight outcomes in later assessments.

Baseline (*T*_b_), midterm (*T*_m4_ and *T*_m8_), end of treatment (*T*_f_), and follow-up (*T*_fu6_ and *T*_fu12_) data on primary and secondary outcomes will be compared between the two groups. Repeated measures gathered for the intervention group during the feedback messaging system will be used to study the effect of behavior and psychological outcomes on weight. Repeated assessment enables testing of the course of relevant factors and their relation to weight.

Moderators and nonspecific predictor variables that can affect outcomes without stemming from the intervention are assessed at baseline (*T*_b_); these variables include pre-surgery body mass index, type of surgery, sex, severity of general psychopathology, and frequency of maladaptive behaviors.

### Statistics

Pearson’s chi-square tests will be used to analyze significant differences in the proportion of cases with weight regain and in specific outcome variables between the intervention group and the control group. Significant weight regain will be considered when higher than 5 kg. The Kaplan–Meier approach (together with the log-rank test) will be used to answer the main question of efficacy. Repeated-measures analysis of variance (ANOVA) will be conducted to explore differences between the participant groups for different key variables at various time points. The main effect for surgery type will be investigated. Variables such as log-in frequency, task submission, satisfaction with the program, socio-demographics, pre-intervention scores on self-report measures, and time since surgery will be tested as covariates and predictors of outcomes using Cox regression models. Relevant baseline differences will be included as covariates. Hierarchical linear models will be used to investigate different trajectories of relevant variables assessed from *T*_b_ to *T*_fu12._ Hierarchical linear models are the models that best fit unbalanced designs with unequal number of observations (missing data) per subject. Self-report measures (for depression, impulsiveness, eating disorder and eating patterns, body image, and general distress) will be examined by conducting an analysis of covariance to explore interference with weight outcomes. Post-hoc comparisons will be conducted using Tukey’s procedure. Controlled effect sizes will be calculated using the Cohen’s *d* statistic.

### Cost-effectiveness

Cost-effectiveness analysis will be conducted from a societal perspective before, during, and after treatment. The incremental cost-effective ratio will be calculated. Differences between the two intervention groups (control group and intervention group) in direct medical expenses related to the internet-based program include the time spent logged into the program for participants and therapists, the time that participants spend engaging in the activities recommended by the program, and the costs associated with data server maintenance and data storage. The value of the time spent in the program will be calculated by multiplying the time estimates by the median hourly wage of the therapists or of a population similar to the program participants. Time absent from work and contact with psychiatric, social, or general medical services will also be considered. Market values will be considered for this analysis.

Other expenses, such as those relating to the development of the internet application, programming costs, (psychoeducational) content development, and chat software are considered ‘sunk costs’ and will not be included in the analyses because they are not expected to recur with dissemination of the program.

### Blinding and data access

Intervention and assessment are conducted exclusively in the APOLO-Bari program. The psychologist participating in the interactive chat sessions does not have access to the program information submitted by the participant. The statistician conducting the statistical analysis has not been involved in the implementation of the program. Treatment allocation has not been previously disclosed to the statistician.

Only the principal investigator and the statistician will have access to the final trial dataset. This is stated in the information for the participant sheet submitted to the ethical committee approval.

### Safety aspects and ethical considerations

The study is being conducted in accordance with the latest version of the Declaration of Helsinki. Approval from the ethical committees involved (Ethics Subcommittee for Life and Health Sciences, University of Minho; Ethics Committee for Health, Hospital of Braga; Ethics Committee for Health, Hospital Center of São João) were obtained and can be consulted found on the current controlled trials website for our trial registration number. Any trial modifications or the inclusion of new health centers as partners for data collection will be communicated to the ethical committees involved as well as to the trial registration organization. Participation in the research project is entirely voluntary. Participants are informed that their present or future medical treatment will not be affected by their decision to participate or not participate. Participants are informed that they can withdraw from the research project at any time without providing a reason. Participants are assigned a code number to maintain confidentiality. All personal data are stored and handled according to data protection principles. In practice, this arrangement means that the written material will be stored in secure locations, and data stored on the computer will be password protected and encoded (SSL technology) and regularly backed up. Additionally, any publication derived from the personal data will be presented in a way that ensures it is not possible to identify individuals. Communications via internet chat will be protected through encoding (SSL technology).

Our team also shares concerns regarding the identification (by our research team or internet program) of patients who might be under extreme distress. If so, the program or researcher will generate an alert, and the project coordinator will proceed to refer the patients to additional, more specific care in the medical center. Alerts will be generated if: (a) significant weight regain is experienced (≥10 kg since the beginning of intervention); (b) there are maladaptive eating behaviors for at least 3 months with a frequency of at least once a week. A participant under individual support may continue the program unless otherwise requested by the accompanying specialist.

## Discussion

This study is the first to develop and test an internet-based intervention for long-term monitoring and support of bariatric patients. Although psychological interventions appear to have promising results in improving postoperative outcomes, no previous study has investigated alternative delivery strategies for weight regain prevention tools.

These interventions may play an important role in broadening the therapeutic reach to bariatric patients who would not otherwise have continuous support. An internet-based program format will overcome difficulties associated with the systematic assessment of health services and the addition of precise or additional guidance, clinical support, and a psych-educational program adapted to address the specificities of this population. Thus, APOLO-Bari is aimed at narrowing the gap between clinical services and patients in a cost-effective format, giving long-distance support to a large number of patients simultaneously, and allowing the allocation of intensive treatment for those in need. Furthermore, this project enables the collection of repetitive data and the study of fluctuations in the risk behaviors that precede weight regain. Targeting these variables in advance could be key for weight regain prevention.

As an important limitation of this study, internet access may not be evenly distributed across the bariatric population, particularly for those with lower socio-economic and educational levels. However, in 2014, 65 % of Portuguese citizens used the internet in the past 3 months, and approximately 58 % access the internet regularly, at least once a week [42]. Furthermore, in 2013, approximately 42 % of the Portuguese population used the internet to seek health information, with rates likely to increase in the following years [43]. Although a growing number of individuals have regular internet access, we provide explanatory sessions in introducing the program, and most patients report that they are able to use such a simple and intuitive program.

Additionally, the self-reporting of weight is acknowledged as an important limitation [32]. Nonetheless, for the study of weight regain and weight loss as the primary outcome of our intervention, weight will be measured by the research team. Furthermore, intervention group participants will be instructed on how to measure their weight and advised not to provide information if their weight was not measured as instructed.

With variable outcomes, particularly long-term outcomes, systematic monitoring and long-term interventions may be critical to the long-term maintenance of weight loss after surgery. In parallel, developing cost-effective interventions to promote adjusted lifestyles and to prevent weight regain appears to be a reasonable approach. The APOLO-Bari study will investigate the role of a comprehensive program as an adjunct intervention to support and monitor patients in the long term following bariatric surgery.

Over the past decade, the digital revolution has provided novel ways for individuals to search for information and communicate. In fact, the digital age has led to alternative ways of accessing health information, monitoring and improving health, and communicating with healthcare partners [44]. Although the digital health revolution has shed light on alternative ways of delivering treatments, further research is needed to investigate their benefits and limitations. This project will add to the field of digital health by testing an adjunctive strategy to enhance weight loss outcomes of bariatric surgery in severely obese individuals and thus bridge the gap between patients and clinical settings. The results of this investigation may greatly affect the outcomes of surgical treatment for severe obesity and have important implications for public health treatments.

## Trial status

This is an ongoing trial that has not completed recruitment of patients.

## Abbreviations

ANOVA: analysis of variance
SSL technology: Secure Sockets Layer
*T*_b_: baseline
*T*_m4_: 4-month midterm assessment
*T*_m8_: 8-month midterm assessment
*T*_f_: final (end of treatment) assessment
*T*_fu6_: 6-month follow-up assessment
*T*_fu12_: 12-month follow-up assessment
